# The Effects of Spinal Cord Stimulators on End Organ Perfusion: A Literature Review

**DOI:** 10.7759/cureus.7253

**Published:** 2020-03-12

**Authors:** Harneel S Saini, Mina Shnoda, Ishveen Saini, Matthew Sayre, Shahzaib Tariq

**Affiliations:** 1 Neurology, Allegheny General Hospital, Pittsburgh, USA; 2 Internal Medicine, Allegheny Health Network, Pittsburgh, USA; 3 Internal Medicine, Lake Erie College of Osteopathic Medicine, Erie, USA; 4 Internal Medicine, Lewis Katz School of Medicine at Temple University, Philadelphia, USA; 5 Neurology, Allegheny Health Network, Pittsburgh, USA

**Keywords:** scs, spinal cord stimulator

## Abstract

Spinal cord stimulators (SCS) have been gaining momentum in the last decade as their role in the management of chronic pain has become more apparent. Our intention was to search, analyze and highlight the effects of spinal cord stimulators on end-organ perfusion. We also looked at vascular diseases of atherosclerotic and nonatherosclerotic nature by examining objective evidence of improved circulation, pain control, limb salvage, and quality of life. We paid specific attention to disease processes such as cerebral hypoperfusion, Chronic-Critical Limb Ischemia, Intractable Angina Pectoris (IAP), Raynaud’s syndrome and Thromboangiitis Obliterans.

We performed a Medline database search for medical literature relevant to Spinal cord stimulators encompassing the years 1950 to 2019. Search terms included “Spinal cord stimulator,” plus one of the following search terms: vasculopathy, stroke, cerebral blood flow, angina pectoris, diabetic ulcers, chronic critical leg ischemia, thromboangiitis obliterans and peripheral vascular disease. We included both clinical and experimental human studies that investigated the effect of SCS’s on end-organ perfusion. We also investigated the pathophysiological mechanism of action of SCS’s on the vasculature. We found 497 articles of which 43 more relevant and impactful articles investigating the hemodynamic effects of SCS and its possible mechanism were selected. Animal studies were excluded from the literature review as they provided heterogeneity. In addition to reporting literature supporting the use of stimulators for currently FDA approved uses, we also actively looked for potential future uses. Spinal Cord stimulators showed improvement in cerebral blood flow, increased capillary recruitment, and better quality of life in many studies. Patients also had increased exercise capacity and a significant reduction in the use of narcotic drug use and daily anginal attacks in patients suffering from IAP.

## Introduction and background

Spinal cord stimulators (SCS) have been gaining more attention in recent years for their ability to help control pain. Currently, the FDA has approved SCS for failed back surgery syndrome (FBSS), chronic painful peripheral neuropathy, multiple sclerosis, complex regional pain syndrome, postherpetic neuralgia, and phantom limb pain. The FDA is currently looking into end-stage PVD and refractory angina as there is strong literature support flowing in from countries abroad. Nonetheless, some insurance companies within the United States have already begun to cover SCS for these disease processes as off-label treatments [1].

A Spinal cord stimulator is an implantable neuromodulation device that sends electrical currents to select areas of the spinal cord for the treatment of chronic pain. It consists of an electrode lead, extension cable, pulse generator, and programmer. There are two types of leads, percutaneous and paddle, and each have their own advantages and disadvantages. Percutaneous leads can be used for the initial screening trial and be permanently placed but have a disadvantage of lead migration. On the other hand, paddle leads are implanted via partial or complete laminectomy and provide more stability and less risk of migration. The electrode is connected to an internal pulse generator which is either of the non-rechargeable or rechargeable type and is programmed by an external transcutaneous telemetry device. Patients have the liberty to turn on/off the device and control the stimulation amplitude and frequency to better aid in pain relief [1-2].

Records dating as far back as 130 AD when Roman physicians would place the black torpedo fish across the painful site and let it discharge electricity until the pain was relieved. Towards the early 20th century, 1919, the first electrical stimulator known as the Electreat was first introduced and designed for therapeutic use by Dr. Charles Willie Kent. Gaining interest, in 1965, the Gate Control theory by Melzack and Wall painted a fundamental concept of how the stimulators worked to provide analgesia. They proposed that nerves responsible for carrying pain and touch/vibratory sensation terminated in the posterior dorsal columns of the spinal cord. From there, a “gate” that could be acted upon by both non-nociceptive signals and nociceptive signals could allow for or deny the passage of the pain signal to make it to the central nervous system. Finally, two years later in 1967, Shealy et al published a case study of the first implanted SCS and its relief of chronic pain in their patient. Further, they also described a difference between acute and chronic pain stating the patient had relief from his chronic pain; however, he retained the ability to sense pinprick stimuli. This further lent support to the gate control theory of pain and SCS’s use in treatment [2]. Shortly after, Dr. Barolat changed the name from the dorsal column to spinal cord stimulators because studies showed better pain control when placed dorsally but in the epidural space rather than on the dorsal columns. Currently, the understanding of SCS work is not completely understood, but according to the Gate Control Theory, stimulation of large non-nociceptive myelinated fibers of peripheral nerves inhibits the activity of smaller nociceptive fibers in the dorsal horn of the spinal cord [3].

The spinothalamic tract and the dorsal column tract both enter the spinal cord through the dorsal horn which carries afferent large-diameter, myelinated fibers for proprioception, discriminative touch and vibration called AB fibers. It also carries smaller myelinated and unmyelinated fibers for pain and temperature. Turning on the stimulator activates the AB fibers inhibiting the radicular pain by activating the A-delta and C fibers respectively [2]. In animal studies, SCS promoted the activation of GABA-ergic and adenosine receptors and the release of GABA, Substance P and serotonin which modulate pain [3]. The pathophysiology of SCS and increased microcirculatory perfusion are currently under research, but our limited understanding revolves around the activation of vasoactive substances. Stimulation of AB fibers by SCS is thought to cause cell signaling molecules like extracellular signal-regulated kinase (ERK) and protein kinase B (AKT) to be activated, which both stimulate TRPV1 (transient receptor potential cation channel subfamily V member 1, also known as vanilloid receptor 1). This in turn depolarizes nerve terminals and releases vasoactive substances like calcitonin gene-related peptide (CGRP) which are known to have potent microvascular vasodilatory properties. CGRP causes endothelial cells to release Nitric oxide (NO) and stimulates blood vessel smooth muscle relaxation, thus causing a decrease in vascular resistance [1-3].

Another theory of mechanistic action is known as the sympathetic response theory. The idea behind this is that SCS decreases efferent sympathetic activity, thus decreasing alpha-1 activity in the vasculature and causing the release of vasoconstriction. The literature currently sites both working in concert to ultimately cause an increase in lower extremity blood flow, while at the same time suppressing nociceptor signals to the brain, all resulting in better pain and symptom control [1-3]. Since then, many studies have demonstrated a benefit of using SCS for patients who suffer from chronic limb ischemia. In this review, we will examine the current literature on the currently FDA-approved uses as well as the off-label and potential uses of SCS and the benefits SCS has on end-organ perfusion.

## Review

Cerebral blood flow

Many experimental models have widely investigated the effects of SCSs on cerebral blood flow (CBF) and found that placement of the stimulator on the cervical spine has can increase the CBF [4]. For many of the studies, the aim was to highlight the effects of SCS on CBF in different intracranial pathological settings such as subarachnoid hemorrhage, stroke, brain injury, and brain tumors. One such retrospective study evaluated 18 patients having various intracranial pathologies such as internal capsule hemorrhage, cerebral ischemia, and deep brain ischemia that had undergone cervical placement of SCS at their institution. They used single-photon emission computerized tomography (SPECT), Near-Infrared Spectroscopy (NIRS), and Transcranial Doppler (TCD) to analyze cerebral blood flow before and after stimulation from the SCS. They found increased CBF with the stimulators turned on with improvement of voluntary movement and dysphagia, decreased spasticity and clonus confirmed by Electromyography (EMG). They reported that no patients worsened after the implant and SCS may offer a possible preventative role in vasospasm and cerebral ischemia [5]. Another interesting study reported positive outcomes in favor of SCS at the cervical versus thoracic level as it increased CBF when placed cervically [6]. A very interesting study evaluated 31 patients in either a vegetative state (VS) or minimally conscious state (MCS) due to a head injury, cerebrovascular accident (CVA), anoxic brain insult or encephalomyelitis. The patients underwent placement of SCS. SPECT showed increased CBF diffusely in the brain, except at the lesion site, and the average CBF of the entire brain increased by 22.2% during stimulation. Three out of the 21 VS patients and 7 out of the 10 MCS patients showed recovery of consciousness. The study could not demonstrate definite evidence that the SCS was useful for the recovery of VS patients, but the study suggested that cervical SCS therapy is effective for MCS patients [7]. High-quality evidence currently lacks in the evaluation of sympathetic modulation of CBF and further studies are warranted ;however during intracerebral vasospasm, a decrease in sympathetic tone might be a potential preventative and therapeutic treatment [6-8].

Intractable Angina Pectoris

The use of spinal cord stimulation (SCS) for Angina was first described in 1987 [9]. Since then, it has proven to be an effective and safe treatment option for this patient population [10]. Spinal cord stimulation (SCS) is currently a Class IIb recommendation by The American College of Cardiology and American Heart Association guidelines for the management of chronic stable angina [11]. In addition to the approved use of Spinal Cord stimulation (SCS) in management of refractory angina, it has now been suggested to have myocardial protective and anti-arrhythmic effect at periods of myocardial ischemia, given their ability to modulate sympathetic activity which plays major role in Heart failure Dynamics as well as pathophysiology of ventricular arrhythmias [12-13]. The Efficacy of SCS in reducing angina symptoms has been supported by a placebo-controlled study, two larger randomized controlled trials and several small controlled studies [14-17]. Hautvast et al. examined SCS in patients suffering from intractable angina pectoris (IAP) by assessing exercise capacity, frequency of anginal attacks, nitrate tablet consumption, and quality of life on 25 patients for 6 weeks. When compared to the control group, the SCS group had an increased exercise duration to angina onset time, better quality of life, and a decreased frequency of anginal attacks and sublingual nitrate consumption. ST depression on exercise EKG also had decreased ischemic episodes [15]. Another study evaluated SCS and intractable angina pectoris by measuring myocardial blood flow (MBF) in 15 patients using positron emission tomography (PET) scans. The mean value of MBF increased from 0.72 to 0.8 ml/min when the stimulator was turned on. Fifty eight regions out of the 75 total regions of the myocardium that were studied had low basal MBF which increased from 0.45 to 0.56 ml/min while 17 of the 75 regions that had high MBF decreased from 1.22 to 1.13 ml/min, thus objectively quantifying that SCS provides an anti-ischemic effect by redistributing MBF [18].

The Electrical Stimulation versus Coronary artery Bypass surgery (ESBY) study compared Spinal cord stimulation (SCS) to Coronary artery bypass grafting (CABG) in high surgical risk. Patients with no prognostic or mortality benefit from Coronary artery bypass grafting (CABG). Both groups showed similar results regarding symptom relief, assessed by a decrease in anginal attack frequency and use of short-acting nitrates. The Coronary artery bypass group (CABG) group performed better on exercise tests with less ischemic ST changes on stress EKG. On the other hand, the CABG group had higher mortality and cerebrovascular morbidity compared to the spinal cord stimulator (SCS) group [16]. A couple of systematic reviews have also shown a benefit for Spinal cord stimulation (SCS) in reducing the frequency of anginal attacks as well as short-acting nitrates consumption. They also have shown that the treatment appears to be safe at both the short- and long-term follow up [10, 19-20]

Peripheral vascular disease

According to the CDC, peripheral vascular disease (PVD) is a rising concern as the number of patients affected by the disease process is increasing. Currently, it is estimated that over 200 million people worldwide and 8.5 million people in the United States alone are affected, with a prevalence as high as 20% in individuals over 60 years old. The presentation of PVD ranges from asymptomatic to debilitating as a progressive disease continuum with an estimated general population awareness of 25% [21-22]. PVD can manifest as distal episodic or progressive occlusion of both, arterial and venous vasculature. In the case of organic PVD such as arteriosclerosis (one of the most common causes of PVD), the distal blood vessels become narrowed by calcification and plaques, thus decreasing blood flow. On the other hand, in functional PVD such as Raynaud's and Breguars, the distal blood vessels become narrowed due to stress, temperature and environmental causes [22-23]. In either case, organic or functional, the pathophysiology is the same; an increase in tissue metabolic demand without adequate compensation leads to tissue damage. If left untreated, these occlusive vascular diseases can manifest as gangrenous limbs, severe systemic infections, and even death. The current treatment of PVD ranges from conservative treatment aimed at symptom relief to amputation.

Non Reconstructable Chronic Critical Leg Ischemia

On the latter, more severe end of the disease spectrum of organic PVD is chronic critical leg ischemia (CCLI), which is characterized by severe ulceration of distal extremities and debilitating ischemic pain even at rest. In 2007 the TransAtlantic Inter-Society Consensus for the Management of Peripheral Arterial Disease (TASC II) updated the guidelines of peripheral arterial disease (PAD) stating that the term critical leg ischemia (CLI) includes “all patients with chronic ischemic rest pain, ulcers or gangrene attributable to objectively proven arterial occlusive disease” (ankle pressures <50 mmHg, toe pressures <30 mmHg and reduced supine forefoot TcPO_^2^_ <30 mmHg), also implying disease chronicity and differentiating it from acute limb ischemia. The study also noted 5-10% of asymptomatic PAD patients will progress to CLI within 5 years and of those patients presenting with CLI, 30% will undergo amputation and another 25% will have died within 6 months [24-25]. The aforementioned definition of CCLI coincides with Fontaine stages III and IV in regards to severity staging of PAD (Table 1). To further establish the severity of this disease having gone untreated, Aquino et al. in 2001 published a study involving 1244 patients afflicted with PAD with episodic claudication over a 15 year period, revealing a decline in ankle-brachial index (ABI) of 0.014 per year with a cumulative 10-year risk of developing ischemic ulcers and pain without exertion as 23% and 30%, respectively [26]. In patients that are not candidates of surgical revascularization or Non Reconstructable Chronic Critical Leg Ischemia (NR-CCLI), the only means of treatment we have to offer currently is conservative with symptomatic control. Medications such as analgesics, vasodilators, and anticoagulants offer a dose-limited relief with amputation as the last resort. However, recent studies involving alternative treatment options with SCS have shown to not only reduce pain but also improve distal circulation and resolve ischemic ulcers without amputation [21-23].

**Table 1:** Fontaine Stage Table for PVD

Fontaine Stage	Symptoms
I	Asymptomatic
II	Intermittent claudication with no pain at rest
III	Pain at rest
IV	Ischemic ulcers, necrosis of tissue

A 2013 Cochrane meta-analysis included 6 international studies (five Randomized controlled trials (RCTs) and one Controlled clinical trial (CCT)) for evaluating the efficacy of SCS in NR-CCLI patients. The meta-analysis included all data in accordance with the TASC II criteria of CCLI and non-reconstructability at the discretion of the treating physician. However, the Cochrane article excluded the functional types of PVD such as Raynaud's and Breugar’s disease as well as milder forms of organic types of PVD such as intermittent claudication. The analysis compared SCS patient groups to conservatively managed patient groups (vasodilators, prostaglandins, analgesics, and anticoagulants), looking primarily at limb salvage and secondarily at pain relief, wound healing, SCS complications, quality of life, and costs. According to the Cochrane analysis, “all studies showed a trend towards a better amputation-free salvage in the SCS group” as low as p=0.08 in the Spincemaille et al. study [25,27].

Subgroup analysis in patients selected by initial TcPO_2_ (Oxygenation) relative to the overall group showed a stronger trend in limb salvage with p values of 0.17 versus 0.47, respectively as in the ESES study [25,28]. The meta-analysis concluded with reporting the Swedish study’s 18-month amputation rate in regards to limb salvage was lower in the SCS group with a p-value of 0.045 and a Number needed to treat (NNT) of 9 to prevent one more major amputation according to the German study [25,28-30]. Looking at secondary outcomes, Cochrane reported the clinical improvement in Fontaine stage 3 to 2 was significantly higher in the SCS group compared to the conservative group (p=0.0014) with an NNT of 3, meaning “three patients have to be treated with SCS for one patient to reach Fontaine stage II (NNT3, 95% CI 2 to 5)” [25]. Both the ESES and German studies included in the Cochrane analysis found that SCS had a better effect on ischemic wound healing compared to conservative management (p=0.013) [25,30]. More objectively, the patients in the SCS group had a 10% increase in ankle-brachial index, while the conservative group had a decrease of 17% (P=<0.02) while some patients (those treated by SCS) had complete resolution of ulcers and increase in ABI of 0.09 (p<0.01) [30]. Lastly, TcPO_2_ measured at baseline was 10mmHg in the SCS group and 12 mmHg in the conservative group. After treatment, TcPO_2 _was found to be significantly better in the SCS group at 21 mmHg and 11 mmHg in the conservative group (p<0.001) [25]. According to the Spincemaille and German studies, pain relief assessed by a visual analogue scale (VAS) was also found to be significantly better at 3 months (p<0.0004) and 12 months (p<0.01) in the SCS group compared to the conservative group; patients in the SCS group also required fewer pain killers compared to the conservative group [25,27,30]. However, Cochrane did report that in amputation vs non-amputation groups pain relief was significantly better in the amputated groups (p<0.01) after statistical analysis [25].

In summary, the Cochrane review entailing studies prior to the year 2003 did not investigate mortality; however, it shed light on the improvement of limb salvage rates and better pain control [25,31]. Another meta-analysis in 2015 aimed specifically at NR-CCLI examined mortality and risk of amputation in 19 studies enrolling 2779 patients found no significant effect on mortality in the groups, but did find a decreased risk of amputation in intermittent pneumatic compression and SCS groups [31]. Interestingly, a retrospective study in 2004 examined 258 patients who had received SCS for organic PVD treatment. The study reported TcPO_2 _values at 18-month post-treatment and found patients with a low TcPO_2_ before SCS treatment (<10mmHg) had limb survival at 77.8% and higher limb survival (89.5%) in patients with a baseline of 10-30 mmHg with sustained TcPO_2_ at 30 mmHg after 18 months in both groups. The study subsequently concluded that “SCS is an effective therapy in improving limb survival in patients with peripheral vascular disease” [32]. A smaller but very promising prospective study in 2011 recruited 40 NR-CCLI patients and followed them up to 12 months after SCS placement at T8-9. The study found 70% increase in supine TcPO_2 _(baseline = TcPO_2_ 19mmHg) and a decrease of 15.3% in TcPCO_2_ in SCS patients (baseline TcPCO2 52mmHg) with a p-value of <0.001. However, more convincing evidence was in the reported dynamic capillaroscopy reading which showed an increase in capillary recruitment (rCBV =0.18mm/sec, p<0.001) and mean capillary density (20 mm2, p<0.0001) post-stimulation. The study further reported an increase in pain relief with no additional painkillers required in 80% of the SCS patients [33].

Thrombangiitis obliterans

**Figure 1:**
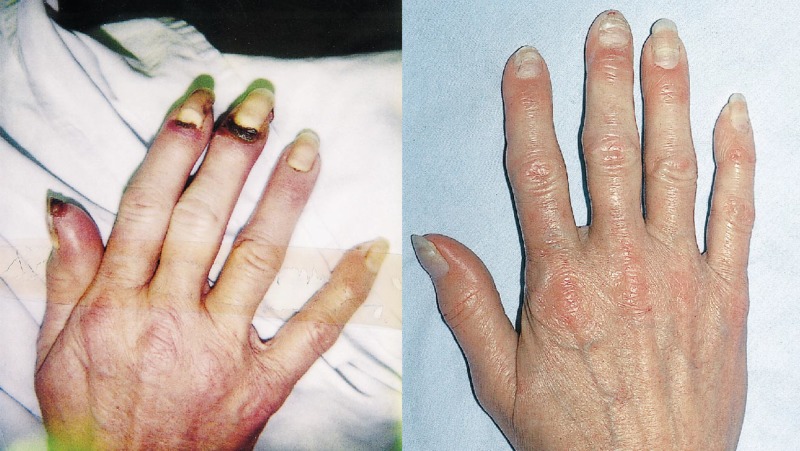
A. Left: Shows patient with ulceration of digits pre-SCS treatment. B Right: Shows complete resolution of ulcers at 1 Month post-SCS implantation and treatment. Picture obtained and published with permission. Picture was obtained and published with permission

Buerger disease or thrombangiitis obliterans (TAO), is a non-atherosclerotic segmentally occlusive inflammatory vascular disease affecting small and medium arteries and veins with a male dominant (3:1) prevalence [34]. This disease is heavily linked to nicotine use and currently is on the decline as more awareness regarding the negative health effects of smoking increases [34-35]. By nature of the disease, it is minimally amenable to revascularization therapy, often causing unrelenting distal limb ulcers and less commonly progression to gangrenous digits. A 2005 retrospective study evaluated the effect of SCS on 29 patients diagnosed with TAO and Raynaud's with a follow up at three months, one, three and five years post-implantation of SCS. The study utilized the regional perfusion index (RPI) which is a ratio between the foot and chest TcPO2. RPI at baseline (before SCS) was 0.27 and increased to 0.41 at three months follow up with a sustained improvement in microcirculation at one and three years of 0.5 and 0.52, respectively. An interesting marked improvement was recognized in a subgroup of 13 patients that had ischemic lesions with an increase from the baseline RPI (0.17) to 0.4 at five years post-SCS, p<0.023 [35]. Another single centered study in Switzerland, in 2013 evaluated 24 patients prospectively and reported a significant increase in TcPO_2_, systolic perfusion via plethysmography and complete limb preservation with resolution of ischemic rest pain in SCS implanted patients who had concurrently decreased tobacco use (<3 cigarettes/day) at three months and four year follow up; the TcPO_2_ was found to be increased in the smoking group from 16 to 40 mmHg (p=0.002) at three months and sustained at 0.4 for four years at the last documented follow up, p=0.003 [36]. A very interesting case report from the Cleveland Clinic reported a 56-year-old woman diagnosed with TAO and after an extensive hospital course, she underwent permanent SCS implantation with complete discontinuation of analgesic therapy and complete resolution of her ischemic digital ulcers (Figure 1) [37].

In our extensive search, we found 4 more case reports highlighting the effects of SCS in a total of 9 patients that met the criteria of TAO that had failed first-line treatment with medications and surgical interventions such as prostaglandin- analouge Iloprost, and bypass grafting. Seven out of the total nine patients among the four reports showed resolution of ulcers, pain control not requiring narcotics, marked improvement in TcPO_2_ and limb salvage [34, 38-40]. The remaining two patients had undergone amputation of the digits with an unclear continued nicotine use [40].

Secondary Raynaud Syndrome and Diabetes Mellitus

**Figure 2:**
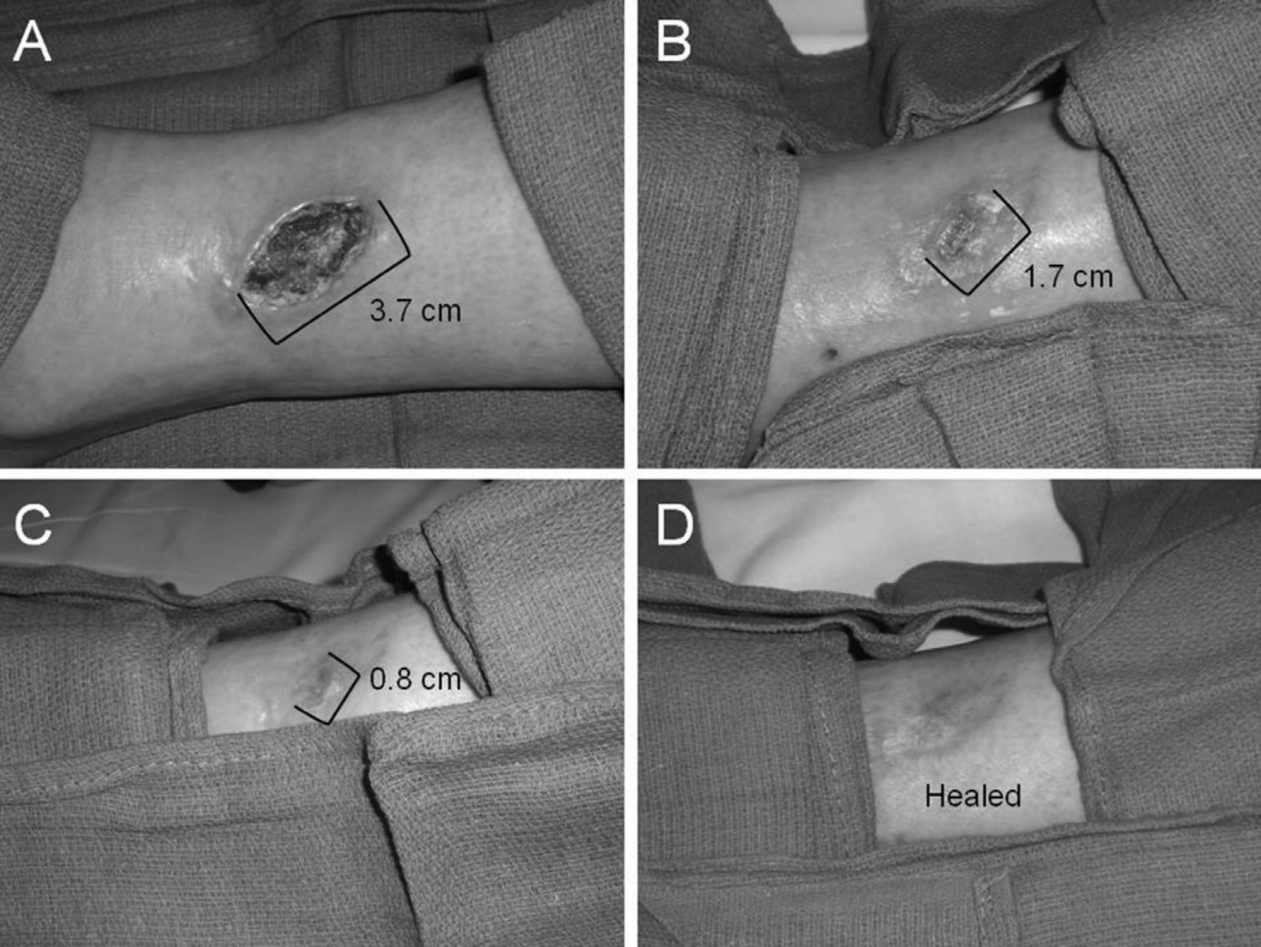
Photographs displaying ulcer size at different time points during healing (A) prior to SCS implantation; (B) 1 month post-implantation; (C) 3 months post-implantation; (D) 4 months post-implantation. Picture was obtained and published with permission

Since the use of SCS is not a gold standard and has been offered to select patients as palliative or alternative treatment, the number of studies on the effect of SCS is limited to case reports in regards to Secondary Raynaud Syndrome (SRS) and Diabetes Mellitus (DM). However, one retrospective single centered study reported increased systolic finger perfusion increased on an average of 25 mmHg and sustained even after four years. The report also documented no ischemic attacks, improvement in the quality of life and pain reduction in the SCS group for SRS patients [34]. One case report presented a 51-year-old female with scleroderma and associated Raynaud’s phenomenon. The patient had a 3.7 cm nonhealing ulcer on the lower extremity with significant microcirculatory insufficiency. After four months post permanent SCS implantation, significant resolution of the ischemic ulcer with sustained (TcPO2 at 40 mmHg) (Figure 2) [41]. Although less studied, SCS has proven to be effective pain control alternatives for diabetic polyneuropathy. One case series evaluating 26 patients prospectively with an average mean follow-up time of four months showed 89% of all neuropathic ulcers healed with minor amputations present in 26% of the cases [42].

Discussion

Spinal cord stimulators have shown great potential in treating end-organ perfusion in various studies and involving various organs, most notably the brain, heart, and integumentary system. The brain is very sensitive to changes in CBF and in order to function optimally, regional and global oxygen demands must be maintained. This is especially important when physiological conditions change in the face of dysautoregulation as seen following an acute ischemic stroke or delayed cerebral ischemia after a subarachnoid hemorrhage causing impaired CBF and progressive structural damage [8]. Furthermore, cerebral hypoperfusion as seen in globally decreased CBF is correlated with cognitive decline and may progress rapidly in patients with dementia and other neurodegenerative diseases [6]. Various animal studies (which were excluded from the review) as well as human studies have shown a global increase in CBF after cervically placed SCS’s were turned on. The consensus among the studies is that SCS may offer a neuroprotective effect for patients suffering from perfusion related brain injuries.

The anti-anginal effect of Spinal cord stimulation (SCS) is less understood and more conflicting compared to their effects in the limbs and brain. Proposed Hypothesis include inhibition of the nociceptive impulse generated from the spinothalamic tract via enhancement of the release of inhibitory gamma-aminobutyric acid (GABA) from the dorsal horn interneurons [43]. The molecular mechanism mediating this effect is suggested to be secondary to changes in the expression of NK-1 receptor, SP and TRPV1 mRNA by Spinal cord stimulation (SCS), which contributes to the attenuation of the cardiac nociceptive afferent signal induced by transient cardiac ischemia [44]. Another proposed hypothesis concludes that Spinal cord stimulation (SCS) causes a reduction in sympathetic tone resulting in a decrease of the coronary oxygen demand [45-47]. Moreover, it causes homogenization of myocardial blood flow by attenuating the effect of dipyridamole or adenosine on coronary arteries. This in turn prevents the deleterious redistribution of blood flow from the subendocardium to the epicardium responsible for inducing the coronary steal syndrome implicated in Anginal pain [47-48]. However, it is important to emphasize that although Spinal cord stimulation can improve clinical symptoms, there is no evidence that it increases coronary blood flow [49-50].

Patients with PVD tend to suffer from symptoms including severe limb pain, claudication and ischemia/necrosis of limbs. Although advances with endovascular surgery have greatly increased diagnostic and symptomatic therapy, there is still a large number of patients who cannot tolerate intervention or are poor surgical candidates. However, even with revascularization or vessel bypass, a lot of these patients will ultimately receive an amputation [39]. In critically debilitated patients, pain relief is essential to living a near-normal life. However, the added effect of treating the underlying ischemic process makes SCS a very promising alternative treatment. Pain killers such as opioids which are commonly prescribed to patients with chronic pain have a notorious effect of inducing tolerance. Patients often need increased amounts of those medications to achieve the same therapeutic analgesic effect as they had once before. This eventually becomes a vicious cycle involving more adverse side effects in consequence of increased pain medication dosages. In the case of opioids, patients may experience nausea, vomiting, constipation, and dependence. Pain relief, as evidenced in the aforementioned studies, was reported as early as one-month post-placement of SCS in patients suffering from Breugars disease, three months for CCL and four months for patients affected by diabetic peripheral neuropathy [25,37,42]. Patients were also prescribed fewer narcotics in the SCS group as compared to the non-stimulator group. This positive response to chronic pain is adequately documented and is currently one of the indications approved by the FDA for SCS placement. However much more research into the exact mechanism is warranted.

Treating the symptoms of patients with end-stage PVD has been the mainstay treatment for many years with amputation, unfortunately, being the end-all and final treatment option for many patients. Interestingly, patients that underwent SCS had a strong trend towards amputation-free salvage of the affected limbs compared to those that were treated with conservative treatment, with many patients having clinical improvement in the ischemic ulcers and improvement from ischemic rest pain to mild claudication [25,27-29]. In treating the underlying pathophysiologic issue of disease progression while providing pain relief, SCS has shown objective and statistically significant evidence of ischemic wound healing through documented increases in transcutaneous oxygen saturations in the distal extremities as well as increased capillary recruitment when compared to those who did not undergo SCS treatment [25,33,37]. Despite the promising results, many of the studies that were presented lacked adequate sample sizes. There simply are not enough randomized studies for CLI patients treated with SCS and there are even fewer randomized studies for patients affected by Breugars, Raynouds, and Diabetes [25]. This very well may be a consequence of a new treatment alternative emerging as SCS’s are not widely used for these disease processes. Specifically, in the patient groups with CLI, one of the inclusion criteria was non-reconstructability of the affected extremity and was left at the discretion of the treating physician. This subjective portion of the inclusion criteria may be responsible for some of the underpowered studies, increasing the likelihood of a Type II error and thus, skewing the results. Regarding the retrospective study from Germany involving patients affected by Breguars, the study failed to report the ABI of non-stimulated patients. Thus, proper conclusions could not be drawn in reference to the effect stimulators have on circulation [35]. Without a proper control group, a proper statistical analysis could be rendered to highlight the efficacy of SCS vs natural progression of the disease. However, anecdotal case reports showing resolution of ischemic ulcers in patients with breugars and scleroderma were quite convincing and further research with randomized control studies is warranted [37,41]. 

Overall, the quality of evidence is low for many of the reported studies worldwide. Nonetheless, some high powered studies have shown to reduce the risk of amputation, and offer an optimal nonsurgical alternative for CLI treatment. In conclusion, SCS has shown a strong statistical trend towards significance in select patients through subgroup analysis. Interestingly, cervical, thoracic or lumbar placement of the SCS has shown to correlate with improvement in disease pathology (Table 2). Early reports researching SCS and their effect on vasculature assessed many of the primary outcomes through a heterogeneous mix of pain scales, ulcer staging criteria and follow-up times. These criteria, at the very least, should be made standard in order to have more reliable data to evaluate the efficacy of SCS on various vasculopathies. Nonetheless, more research is required to better evaluate the cost-effectiveness of SCS versus conservative and surgical treatment for vasculopathy like end-stage PVD. The initial studies have shown decreased narcotic use, increased chance of limb salvage and better quality of life which is a great progress for such end-stage and life-altering diseases. SCS has shown to not only relieve the pain but also help treat the underlying disease process. It is our opinion that further research should focus on a select patient population that would benefit from the therapy the most in order to elucidate the microcirculatory effect of SCS’s. The aforementioned studies have paved the way for SCS as an alternative and nonsurgical treatment for various vasculopathies and are well worth the time to investigate further.

**Table 2:** Spinal Cord Stimulator Placement Coronary artery disease (CAD); SCS: Spinal Cord Stimulator; RCT: Randomized Control Study; CBF: Cerebral Blood Flow; Percutaneous Myocardial Laser Revascularisation (PMR)

Location of SCS	Reference	Type of Study	Year of Publication	Type of Pathology	Brief	Outcome
Cervical						
	Visocchi et al. [5]	Retrospective	2002	Stroke	Evaluated 18 patients with a stroke that underwent cervical SCS placement with particular attention towards CBF. They looked at transcranial Doppler (TCD), SPECT and NIRS. .	SCS showed an increase in regional CBF in 75% of patients through SPECT and showed improvement also in dysphagia, clonus, voluntary movement and endurance with a decrease in spasticity. Albert score showed improvement from 80 to 100 with EMG-confirmed recordings
	Mazzone et al. [6]	Prospective	1996	Pain, spasticity or bladder incontinence	A total of 12 patients with either pain, spasticity or bladder incontinence were evaluated for regional CBF with cervical or thoracic SCS placement .	Patients that underwent cervical SCS placement had a symmetrical increase in regional CBF in the anterior brain regions of over 70% of the patients relative to the patients that underwent thoracic SCS placement.
	Yamamoto et al. [7]	Prospective	2017	vegetative state and minimally conscious state from TBI	Evaluated 21 vegetative state (VS) and 10 minimally conscious state (MCS) patients with cervically placed SCS for electrophysiological changes 3 months after the onset of TBI.	14% of VS and 70% of MCS recovered consciousness. after 12 months of SCS treatment with preserved fifth wave. 5Hz cervical stimulation caused increased cerebral blood diffusely by 22.2 % (p<0.0001)
Thoracic						
	Taylor et al. [10]	Systematic Review	2009	CAD	total of 270 patients with refractory angina patients, comparing outcome between receiving SCS and CABG or percutaneous laser revascularization.	SCS has similar efficacy and safety when compared to percutaneous laser revascularization in patients with refractory angina.
	de Jongste et al. [14]	RCT	1994	CAD	Assessment of exercise capacity via treadmill exercise and Quality of life assessed by daily and social activity scores in patients with chronic angina assigned to SCS vs medical treatment only.	SCS group showed significant improvement in exercise capacity and quality of life
	Hautvast et al. [15]	RCT	1998	CAD	Daily frequency of anginal attacks and use of nitrates tablets in patients with intractable angina in SCS group compared to medical treatment only group.	Frequency of Anginal attacks and sublingual nitrate consumption are reduced in the SCS group
	Mannheimer et al [16]	RCT	1998	CAD	Patient randomized into CABG or SCS and the patients assessed in 6 months with respect to symptoms, exercise capacity, ischemic EKG, change during exercise.	CABG and SCS groups had equivalent results in terms of symptoms relief, However, CABG group had an increased exercise capacity and less ST-Segment depression on maximum workloads.
	McNAb et al [17]	RCT	2006	CAD	Patient with angina CCS class 3/4 randomized to receiving either SCS or PMR, primary outcome was comparing exercise treadmill time on modified bruce protocol over 12 months.	little evidence of difference in effectiveness between SCS and PMR when comparing both groups angina free exercise capacity
	Lanza et al. [19]	Systematic Review	2012	CAD	systematic review of observational studies of the effect of SCS in patients with with refractory angina published in the time period between 1987 and 2010	Consisted of reduction in the number of anginal attacks and consumption of short-acting nitrates.
	Hautvast et al. [48]	RCT	1996	CAD	myocardial blood flow studied by PET before and after SCS both at rest and after dipyridamole stress	myocardial flowheterogeneity is reduced after SCS insertion both at rest and after dipyridamole stress
	Fricke et al. [49]	RCT	2009	CAD	Coronary flow reserve assessed using PET scan at the start of the study and 1 year after using SCS	Patients had relief of anginal symptoms however with no change in CFR in 1 year post SCS placement.

## Conclusions

Spinal Cord stimulators have a well-documented and long history for their analgesic properties. SCS’s have an already established role in patients suffering from chronic pain and evidently have potential in healing vasculopathies. We believe SCS have a place as an alternative and nonsurgical treatment for many vasculopathies; however, while we realize that SCS may not be applicable to everyone, SCS definitely has a niche in a subset of patients on the later more severe side of end stage vasculopathy in which small changes in microperfusion can help immensely. This population of treatable patients is yet to be identified and future research should aim towards more suitable patients. A larger sample and randomized clinical trials are needed in order to compare the efficacy of spinal cord stimulators to sole medical management as well as other modalities in refractory angina. So far, Spinal cord Stimulators use is only proven to relieve Anginal symptoms; more Clinical trials targeting their ability to affect coronary blood flow/reserve are needed. Studies targeting Spinal cord stimulators in management of Heart failure (HF) and ventricular arrhythmias are very limited and mostly on animals. More trials are needed to establish if they can be used in patients with Heart failure or in those at risk for developing ventricular arrhythmias. Finally, the cost and availability of Spinal cord stimulators remain an issue and more studies targeting the cost-effectiveness of their use in the target population are recommended. The effect of SCS on CBF is relatively new and requires more investigation; however, many preliminary studies have shown a potential benefit to a select patient population. Spinal cord stimulators have a potential microcirculatory benefit for select patients suffering from the aforementioned vasculopathies. However, we believe further high-quality research is needed and should be directed towards the cost-effectiveness of administering conservative approaches versus spinal cord stimulators as an alternative treatment option.
